# Quality Evaluation of Methyl Binding Domain Based Kits for Enrichment DNA-Methylation Sequencing

**DOI:** 10.1371/journal.pone.0059068

**Published:** 2013-03-15

**Authors:** Tim De Meyer, Evi Mampaey, Michaël Vlemmix, Simon Denil, Geert Trooskens, Jean-Pierre Renard, Sarah De Keulenaer, Pierre Dehan, Gerben Menschaert, Wim Van Criekinge

**Affiliations:** 1 Department of Mathematical Modelling, Statistics and Bioinformatics, Faculty of Bioscience Engineering, Ghent University, Ghent, Belgium; 2 Department of Internal Medicine, Faculty of Medicine and Health Sciences, Ghent University, Ghent, Belgium; 3 MDX Health S.A., Ghent, Belgium; 4 Department of Experimental Pathology, University of Liege, Liege, Belgium; University of Bonn, Institut of experimental hematology and transfusion medicine, Germany

## Abstract

DNA-methylation is an important epigenetic feature in health and disease. Methylated sequence capturing by Methyl Binding Domain (MBD) based enrichment followed by second-generation sequencing provides the best combination of sensitivity and cost-efficiency for genome-wide DNA-methylation profiling. However, existing implementations are numerous, and quality control and optimization require expensive external validation. Therefore, this study has two aims: 1) to identify a best performing kit for MBD-based enrichment using independent validation data, and 2) to evaluate whether quality evaluation can also be performed solely based on the characteristics of the generated sequences. Five commercially available kits for MBD enrichment were combined with Illumina GAIIx sequencing for three cell lines (HCT15, DU145, PC3). Reduced representation bisulfite sequencing data (all three cell lines) and publicly available Illumina Infinium BeadChip data (DU145 and PC3) were used for benchmarking. Consistent large-scale differences in yield, sensitivity and specificity between the different kits could be identified, with Diagenode's MethylCap kit as overall best performing kit under the tested conditions. This kit could also be identified with the Fragment CpG-plot, which summarizes the CpG content of the captured fragments, implying that the latter can be used as a tool to monitor data quality. In conclusion, there are major quality differences between kits for MBD-based capturing of methylated DNA, with the MethylCap kit performing best under the used settings. The Fragment CpG-plot is able to monitor data quality based on inherent sequence data characteristics, and is therefore a cost-efficient tool for experimental optimization, but also to monitor quality throughout routine applications.

## Introduction

DNA-methylation is an epigenetic process associated with gene expression regulation. In mammalian somatic cells, it predominantly occurs at cytosines in a CpG-dinucleotide context, and it is catalyzed by DNA-methyltransferases. In the human genome, up to 80% of CpGs have been reported to be methylated. While this appears to be a fixed status for most CpGs, the methylation degree of longer stretches of DNA enriched in CpG-dinucleotides, i.e. "CpG-islands", is more versatile and particularly associated with transcriptional regulation, e.g. in cellular differentiation, imprinting of paternal/maternal alleles and female X chromosome inactivation [1], [2]. Particularly when located in promoter or first exon regions, CpG-island methylation has been reported to lead to transcriptional silencing [3]. Aberrant DNA-methylation has been associated with a plethora of diseases, including most types of cancer, cardiovascular disease and Alzheimer's disease [4], [5], [6]. In these and other diseases, DNA-methylation studies have led to the identification of novel biomarkers and risk factors, with clinical applications in the diagnostic, prognostic and pharmacogenomics fields [7], [8], [9], [10], [11].

Until recently, applications were predominantly limited to locus specific methylation assays, for examples see references [9], [12], but the advent of high-throughput technologies has introduced the possibility of genome-wide DNA-methylation profiling. Most methodologies are based on the application of bisulfite treatment to genomic DNA, which chemically converts unmethylated cytosines to uracil, but leaves methylated cytosines intact. Uracil corresponds to thymine with respect to its basepairing behavior. After sequencing, DNA-methylation status differences are implied by sequence differences as only unmethylated cytosines will be observed as thymines [13]. Whole genome sequencing of bisulfite treated DNA is possible, but as sufficient coverage over the full genome should be obtained for quantification, costs are currently even higher than for normal whole genome sequencing. Methods such as reduced representation bisulfite sequencing (RRBS) [14] therefore reduce the proportion of the genome to be sequenced. Alternatively, relatively inexpensive bead array-based methods have been developed for bisulfite treated DNA (Illumina Infinium BeadChip) [15]. Besides the traditional array problems, even though the novel 450k Infinium BeadChips covers a major part of the human DNA-methylome [16], the genome-wide character and quality of these assays are inherently limited by the probe design.

The major alternative for bisulfite treated DNA-characterization is the purification of methylated DNA-fragments followed by sequencing, which allows for a cost-efficient (order of magnitude, 10^3^ €), genome-wide approach. Although there are several possibilities with often complementary strengths [17], specific antibodies for methylated DNA immuno-precipitation (MeDIP) [18], [19] are most widely used. However, the application of methyl-CpG binding domain (MBD) proteins [20], [21] for affinity based purification is believed to be inherently better due to the biological origin of the MBD. A recent study demonstrated that, when total coverage is sufficient, MBD-seq (also called MethylCap-seq or MiGS) is generally more sensitive than MeDIP-seq [22], [23] and methylation specific microarrays (after bisulfite treatment) [22]. A greater sensitivity for MBD compared to MEDIP was also confirmed in a microarray based study [24]. Therefore, until further optimization of sequencing technologies allows for a cost-efficient whole-genome sequencing of bisulfite treated DNA or direct detection of methylated cytosines at base-resolution, MBD-seq might easily become the most widely used methodology.

Lately, several commercial ‘DNA-methylation capturing’ kits for MBD-based affinity purification have been developed, typically with different options regarding salt concentration for the elution step. For an optimal analysis it is important to use the most sensitive and specific methodology available. However, independent information about the yield, specificity and sensitivity of these kits (and the different options) is completely absent, and the only manner to assess this is to use expensive external validation, e.g. by bisulfite sequencing. In addition, there are no objective measures to identify aberrant profiles which should be excluded from further analysis. Indeed, while MBD-seq is rapidly gaining importance, there is a need for a straightforward quality evaluation tool, cf. the diagnostic plots for microarrays e.g. [25]. The Fragment CpG-plot, depicting the CpG content of the captured fragments, has the potential to be such a tool.

Therefore, we evaluated five commercially available MBD-based DNA-methylation purification kits for combination with next generation sequencing, and results were benchmarked by two sets of data generated using other platforms (RRBS and Infinium HumanMethylation27 BeadChips). In order to evaluate the consistency of the results, conclusions were compared between three different cell lines (HCT15, DU145 and PC3). Subsequently, it was assessed whether the same conclusions could be obtained based solely on the Fragment CpG plot.

## Materials and Methods

### Sample preparation

We used 3 different human cell lines: DU145, PC3 (prostate cancer) and HCT15 (colon cancer). Cell lines PC3 and HCT15 were purchased from ATCC and were collected at passage 3. Cell line DU145 was purchased from CHU Liège and cells were collected at passage 9. Genomic DNA was extracted from these cell lines with the Easy DNA kit (Invitrogen K1800-01) according to protocol #4. The DNA concentration was measured on a NanoDrop ND-1000 (Thermo Scientific, Wilmington, North Carolina, USA).

### DNA fragmentation

Fragmentation of the genomic DNA was performed on Covaris S2 (Covaris, Woburn, Massachusetts, USA) with following settings: duty cycle 10%, intensity 5, 200 cycles per burst during 180 seconds to obtain fragments with an average length of 200 bp. The power mode was frequency sweeping, temperature 6–8°C and water level 12. 500 ng was loaded in 130 µl TE (1∶5) in a microtube with AFA intensifier (Covaris, Woburn, Massachusetts, USA). Length of the fragments was analyzed on a DNA High Sensitivity chip on an Agilent 2100 (Agilent Technologies, Santa Clara, California, USA). Concentration was determined on a FluoStar Optima plate reader (BMG Labtech, Offenburg, Germany) with the Quant-iT™ Picogreen® dsDNA assay kit (Invitrogen P7589, Merelbeke, Belgium) on 480/520 nm.

### Kit selection and methylated DNA capturing

All MBD-based capturing kits commercially available at the time of study initiation were included in this study. Tested kits, with indication of used MBDs (although typically recombinant forms), were: MethylMagnet™ mCpG DNA isolation kit (MBD2b) (Ribomed MM101-K, Carlsbad, California, USA), MethylCollector™ (MBD2b) and MethylCollector™ Ultra (MBD2b and MBD3L1) (Active Motif 55005, Carlsbad, California, USA), MethylCap™ kit (MBD from MeCP2) (Diagenode AF-100-0048, Liège, Belgium), MethylMiner™ Methylated DNA Enrichment Kit (MBD2) (Invitrogen ME10025, Merelbeke, Belgium). For the remainder of the manuscript, these kits are referred to as respectively MethylMagnet, MethylCollector, MethylCollector Ultra, MethylCap and MethylMiner kits. Frequency of use, as estimated by Google Scholar citations (http://scholar.google.com) at the beginning of January 2013, were roughly 50 times for the MethylCollector kits, 25 times for MethylCap and 50 times for MethylMiner, whereas only 3 references to the MethylMagnet kit could be found.

For each cell line, 200 ng of the fragmented DNA was subjected to every kit following the manufacturer's recommended protocol using the highest salt concentration and with the only exception that short (< = 5 min) centrifugation and rotation steps were performed at room temperature (instead of 4°C) for the MethylCap kit. Quantification of the captured DNA was performed with the FluoStar Optima plate reader (BMG Labtech) with the Quant-iT™ Picogreen® dsDNA assay kit (Invitrogen P7589) on 480/520 nm. The eluted DNA was purified using a MinElute Reaction Cleanup kit (Qiagen 28204, Germantown, Maryland, USA).

### Illumina library preparation

As kit yields were often too low for reliable detection (Table 1), for each captured fraction, the complete amount of purified DNA was used for library preparation, which was performed with a modified ‘multiplexed paired-end ChIP protocol’ (Illumina, San Diego, California, USA). The NEBNext® DNA Sample Prep Master Mix Set 1 (New England BioLabs (NEB) E6040, Ipswich, Massachusetts, USA) was used in combination with the Multiplexing Sample Preparation Oligo Kit (Illumina PE-400-1001). For each kit, a different barcode was used for each cell line, implying that observed effects cannot be attributed to different barcode sequencing efficiencies.

**Table 1:** MBD-based kit yield, as physical yield, and as number of raw fragments, uniquely mapped fragments and non-duplicate uniquely mapped fragments after sequencing, for each cell line.

Cell line	MBD-based kit	a. Physical yield (ng)	b. Number of sequenced fragments*	c. Total uniquely mapped fragments (% of b)	d. Non-duplicate uniquely mapped fragments (% of c)
HCT15	MethylMagnet	4.85	1,805,640	734,996 (40.7)	60,194 (8.2%)
	MethylCollector	N/A	3,664,676	1,677,359 (45.8)	266,922 (15.9%)
	MC Ultra	6.30	3,576,381	1,514,976 (42.4)	655,819 (43.3%)
	MethylCap	9.38	11,531,844	6,632,940 (57.5)	3,916,243 (59.0%)
	MethylMiner	24.80	15,315,046	9,759,129 (63.7)	7,387,361 (75.7%)
DU145	MethylMagnet	1.75	155,564	81,179 (52.2)	10,054 (12.4)
	MethylCollector	N/A	230,880	120,948 (52.4)	29,656 (24.5)
	MC Ultra	N/A	146,551	79,066 (54.0)	30,357 (38.4)
	MethylCap	4.30	2,733,079	1,483,445 (54.3)	825,370 (55.6)
	MethylMiner	25.80	6,700,917	4,259,089 (63.6)	2,968,040 (69.7)
PC3	MethylMagnet	2.00	1,500,615	742,693 (49.5)	243,273 (32.8)
	MethylCollector	N/A	277,756	155,694 (56.1)	85,153 (54.7)
	MC Ultra	2.40	1,363,234	1,090,164 (80.0)	580,789 (53.3)
	MethylCap	8.45	2,763,144	1,672,190 (60.5)	952,757 (57.0)
	MethylMiner	48.30	3,103,308	2,179,903 (70.2)	2,102,540 (96.5)

MC Ultra indicates MethylCollector Ultra, and N/A indicates not available due to too low amounts for accurate measurements.

* Due to the paired-end sequencing, one fragment corresponds with 2 reads.

### Library amplification and sequencing

22*μ*l of DNA was subjected to PCR following the Illumina Library Amplification Index Protocol (Illumina) with 21 cycles of PCR amplification. PCR products were purified on Qiaquick PCR Purification columns (Qiagen 28101) and eluted in 50*μ*l elution buffer (1:5). Next, the libraries were concentrated in a rotary evaporator (Jouan 11176740, St-Herblain, France) to 10*μ*l and assessed using an Agilent 2100 High Sensitive DNA chip (Agilent Technologies). The concentration was determined by qPCR with a PhiX index3 standard solution (Illumina PE-400-1002). Only then, libraries were pooled per four (2*μ*l aliquots at 10nM of each individual library), and each of these pools was used for NaOH denaturation. After denaturation, pools were diluted to 10pM and used for sequencing on an Illumina Genome Analyzer IIx following the Illumina protocol: ’performing a multiplexed paired-end run‚ (2 times 45 cycle). As such, sequencing was performed with 4 libraries per lane and one control lane with PhiX index3 control (Illumina PE-400-1002).

### Data processing and the fragment CpG plot

For each cell line and kit combination, paired-end reads were mapped using BOWTIE [26]. Only those fragments that mapped uniquely within a 400 bp of each other in the human reference genome (NCBI build 37) were retained. Here, we define a "mapped fragment" as the reference genome sequence corresponding with a mapped fragment, including both sequenced ends and the region in between. Further data-analysis was performed using R 2.15.0.

The Fragment CpG plot depicts the CpG content of the captured fragments, *i.e.* the frequency of mapped fragments with a certain CpG content. For the creation of this plot, the amount of CpGs was counted for each obtained mapped fragment. Suppose that, for a specific sample (*i.e.* cell line-kit combination), there are *F_i_* fragments with *i* CpGs (*i* = 0, 1, 2,...), yielding a total of Σ*_i_*(*F_i_*) fragments for that sample. The Fragment CpG plot then depicts the normalized *F_i_* counts, i.e. *F_i_*/Σ_i_(*F_i_*), as a function of *i*, for that sample. The R-script for this quality control tool is available upon request.

### Bisulfite sequencing and Infinium BeadChip data

Reduced representation bisulfite sequencing [14] was performed by BaseClear (Leiden, the Netherlands), using the EpiQuest DNA Methylation Analysis Platform (http://www.baseclear.com/dna-sequencing/next-gen-sequencing/epiquest-5-mc-analysis/) yielding 2×50 paired end bisulfite sequence reads, with total coverages of 27.9, 39.2 and 51.0 million paired reads for respectively HCT15, PC3 and DU145 which were further processed using a custom pipeline (BaseClear). For each CpG, intensities for both strands of the genome were summed for comparison with MBD-seq data.

Independent Infinium HumanMethylation27 BeadChip data, as reported by Kim et al. [27], were downloaded from the Gene Expression Omnibus (GEO, accession number: GSE23388, data were already quantile normalized). R-package IlluminaHumanMethylation27k.db and GEO dataset GPL8490 were used to identify the exact location of each CpG assessed by the individual probes. The UCSC liftOver tool (http://genome.ucsc.edu/cgi-bin/hgLiftOver) with standard settings was applied to convert CpG-loci from human reference genome build 36 to build 37, with a success rate of 99.95%.

### Availability of generated data

The data sets supporting the results of this article are available in the GEO repository [GSE42790; http://www.ncbi.nlm.nih.gov/geo/query/acc.cgi?token=bjslvemcuqkysju&acc=GSE42790].

## Results

In the first part of the results section, the yield of the different kits (see Table 1) is assessed as a first indication of kit and experiment quality, followed by an exploratory analysis of the DNA methylation patterns for some loci. The second part consists of an evaluation of sensitivity and specificity of the different kits, using two sources of independent data. Finally, the conclusions from these analyses are compared with those generated by the fragment CpG plot.

### Kit yield evaluation

For each condition (kit and cell line), 200 ng of DNA was used, which is appropriate according to all the manufacturers' instructions and realistic in a clinical setting. Although only indirectly associated with data quality, the yield of a given kit or enrichment methodology is a very important characteristic. The most direct measure to assess yield is to measure the amount of DNA isolated for each condition (kit/cell line combination). However, as these amounts were often too low for accurate measurements (Table 1), we also assessed the number of sequenced fragments. As there will be a major impact of the other libraries sequenced within the same lane, this number can only be considered as a semi-quantitative measure.

The sequencing protocol might result in multiple fragments uniquely mapping on exactly the same location in the genome, and therefore most likely originating from the same sequence (duplicates). Low kit yields associated with high non-duplicate fractions would therefore most likely indicate decreased sequencing (and not kit) efficiency. Low yields associated with low non-duplicate fractions on the other hand clearly indicate the low kit efficiency as the underlying cause: low numbers of fragments were pre-amplified resulting in low numbers of, predominantly duplicate, fragments. Table 1 therefore also summarizes the number of non-duplicate, mappable fragments. Overall, these numbers reflect the directly measured kit yield (in ng), also in Table 1, although it should be noted that the relationship is clearly not linear.

Table 1 demonstrates that there are large differences between cell lines (samples), but even more between kits. The MethylMiner kit resulted in the highest yield, followed by the MethylCap kit. Both MethylCollector kits and the MethylMagnet kit are featured by very low yields when starting from 200 ng input material. It is clear that the different yields are inherent kit characteristics, and will certainly have an impact on data quality: since higher yields might be caused by a higher sensitivity, but also by a lower specificity, these features are evaluated in the next paragraphs. For the remainder of the results section, for each individual condition, multiple fragments mapping to the exact same locations on the human genome (duplicate fragments) were considered as originating from, and further processed as, a single fragment. In a first attempt to assess the sensitivity and specificity of the different kits, the generated DNA methylated patterns are visually inspected for a selected set of loci.

### Exploratory analysis: comparison with RRBS results for selected set of loci

**Figure 1:**
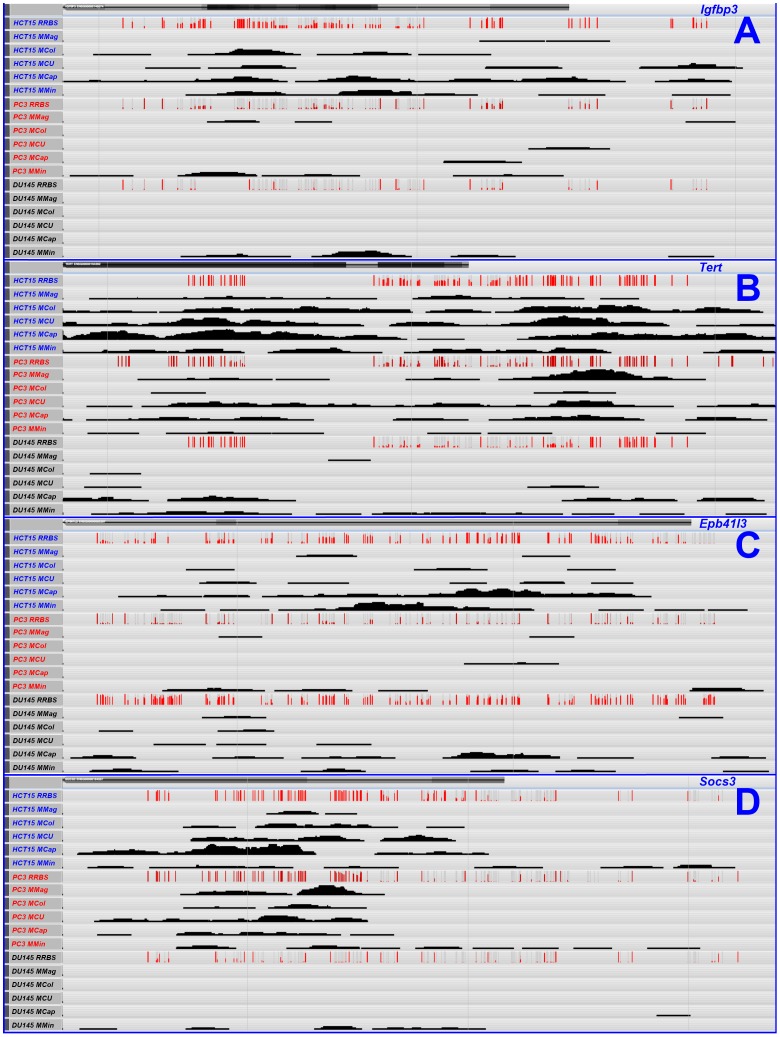
Exploratory comparison of MBD-seq and RRBS data. Visual comparison of MBD-seq results for MethylMagnet (MMag), MethylCollector (MCol), MethylCollector Ultra (MCU), MethylCap (MCap) and MethylMiner (MMin) with RRBS data for the promoter regions of four selected loci, i.e. *Igfbp3* (panel A, chromosome 7, depicted from position 45959883 to 45962146), *Tert* (panel B, chromosome 5, 1293850 to 1296219), *Epb41l3* (panel C, chromosome 8, 5628365 to 5630973) and *Socs3* (panel D, chromosome 17, 76354158 to 76357420). CpGs assessed by RRBS are indicated as vertical red (fraction methylated)/grey (fraction unmethylated) bars. Note that RRBS only assesses the methylation status of a (sometimes variable) subfraction of CpGs.

The DNA-methylation status of the promoter regions of 4 selected loci was compared between the different kits and samples using the RRBS results as gold standard. Figure 1 depicts the MBD-seq (mapped putatively methylated fragments) and RRBS (% methylation) results for *Igfbp3* (panel A), *Tert* (panel B), *Epb41l3* (panel C) and *Socs3* (panel D) for all three cell lines. Figure 1 demonstrates that, in general, MBD-seq adequately detects methylation when a certain number of CpGs is sufficiently methylated. However, there is variation between kits that cannot be solely attributed to yield differences. For example, the MethylMiner kit often suggests presence of DNA-methylation, e.g. in the *Igfbp3* and *Socs3* promoters of DU145, for which RRBS evidence is poor at most, implying low specificity. Also for MethylMagnet, specificity often appears to be low, e.g. in the *Igfbp3* and *Epb41l3* promoters for PC3. The MethylCap and both MethylCollector kits appear to be featured by a reasonable sensitivity and specificity, but yield differences complicate a finer comparison. More advanced, genome-wide analyses are therefore required.

### Genome-wide kit comparison with independent validation data

**Figure 2:**
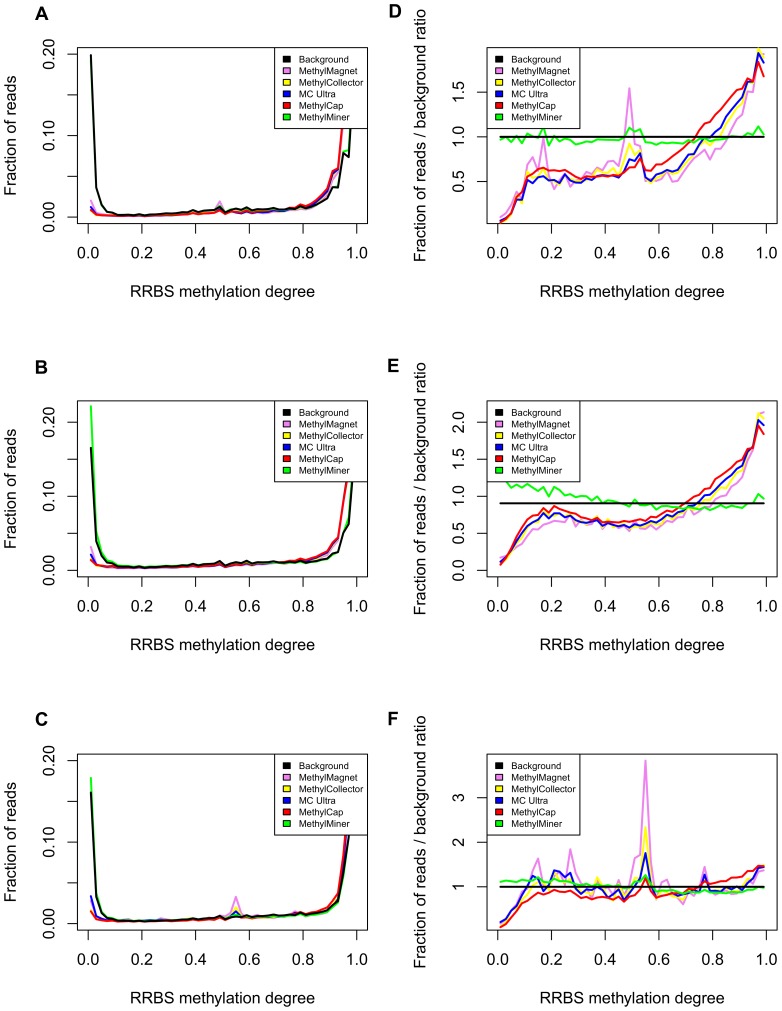
Yield independent genome-wide kit evaluation using RRBS data for external validation. Fractions of mapped MBD-seq fragments corresponding with specific RRBS methylation degrees (binned per 2%) for the different cell lines (A, HCT15; B, DU145; C, PC3) and kits (violet, MethylMagnet; yellow, MethylCollector; blue, MethylCollector Ultra (MC Ultra); red, MethylCap; green, MethylMiner) with indication of the background profile (black, fractions of all RRBS values measured for specific cell line). Additionally, the same fractions after division by the corresponding background profile fractions are plotted (D, HCT15; E, DU145; F, PC3).

MBD-seq based data were compared with RRBS results for the three cell lines. In order to obtain sufficient resolution, only loci with RRBS coverages> = 20 were considered. Direct evaluation of sensitivity and specificity of the kits is complicated by the major yield differences between kits, *e.g.* higher yields are typically associated with higher sensitivity and/or lower specificity. Here, a yield independent approach is envisaged by plotting the *fractions* of mapped fragments corresponding with specific RRBS derived CpG methylation degrees (binned per 2%), e.g. a Y-axis value of 0.03 for RRBS values between 0 and 0.02 for a specific kit implies that 3% of the mapped fragments for that kit contain virtually unmethylated (between 0 and 2%) CpGs as determined by RRBS. As the fractions sum to one for each of the kits, the profiles are independent of the major yield differences. In addition, a background profile is plotted which depicts all loci assessed by RRBS, i.e. the full pool of loci that can be captured by the different kits (Figure 2, A–C). Division by the background profile fractions for the corresponding RRBS methylation degrees further clarifies the result (Figure 2, D–F). Note that the plots are CpG-oriented: fragments may contain several CpGs assessed by RRBS, and might therefore belong to several of the fractions in the plot. However, these dependencies between data points are negligible compared to the massive amounts of data depicted.

Except for MethylMiner, all kits captured only low fractions of alleles featured by absence of methylation, whereas there was a clear enrichment for heavily methylated alleles (Figure 2). Under the evaluated conditions, the MethylMiner profile mimics the background profile, i.e. the high yield for this kit (Table 1) can be explained by the very high fraction of noise captured. It is clear from Figure 2 that the MethylCap and MethylCollector kit exhibit the lowest fraction of fragments with RRBS methylation degrees ≅ 0 and are therefore featured by the highest specificity, followed by the MethylCollector Ultra and MethylMagnet kits. Sensitivity can here be defined as a kit's capacity to capture fragments with lower methylation degrees, and can be assessed as the point where a kit's profile is consistently higher (enriched) compared to the background profile. For all three cell lines, the MethylCap kit appears to be featured by the highest sensitivity. Although less clear for DU145 (lower resolution due to lower yields for several kits, cf. Table 1), the MethylCap kit is followed by the MethylCollector, MethylCollector Ultra and MethylMagnet kits regarding sensitivity (ignoring the MethylMiner profile). Note that the peak heights for RRBS methylation degrees ≅ 1 are less informative as the plotting of fractions implies that more narrow peaks are also typically higher.

**Figure 3:**
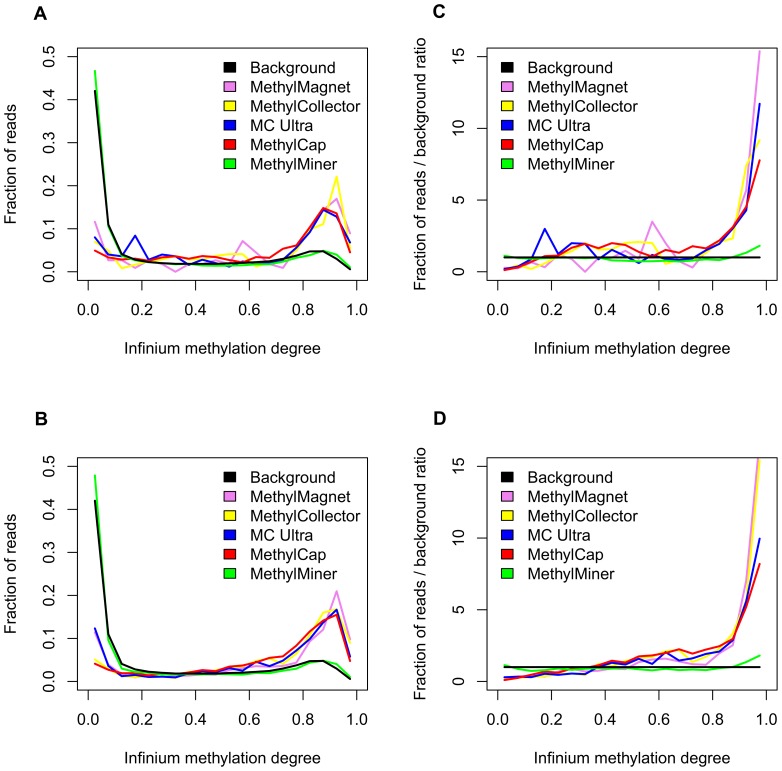
Yield independent genome-wide kit evaluation using Infinium HumanMethylation27 BeadChip data for external validation. Fractions of mapped MBD-seq fragments corresponding with specific Infinium methylation degrees (binned per 5%) for two cell lines (A, DU145; B, PC3) and all kits (violet, MethylMagnet; yellow, MethylCollector; blue, MethylCollector Ultra (MC Ultra); red, MethylCap; green, MethylMiner) with indication of the background profile (black, fractions of all Infinium methylation values measured for specific cell line). Additionally, the same fractions after division by the corresponding background profile fractions are plotted (C, DU145; D, PC3).

As an additional validation step, the MBD data obtained with the different kits were also compared with Infinium HumanMethylation27 BeadChip results, independently generated by Kim et al. [27], for cell lines DU145 and PC3. Here, it should be stated that the use of external methylation data entails the possibility that different experimental conditions between different labs could already have introduced changes in methylation, but that this will have equal effects on each evaluated kit. Similar plots were created as for the RRBS data, but using Infinium beta-values (binned per 5%) as measure for methylation degrees (Figure 3). Overall, very similar conclusions as for the comparison with RRBS data can be made. For these cell lines, the highest specificity could be observed for MethylCap, followed by MethylCollector, MethylCollector Ultra and MethylMagnet, whereas the MethylMiner profile again reveals lack of sensitivity and specificity. The largest sensitivity (profile consistently above background) is again observed for MethylCap. Although the ranking of the other kits is less clear, most likely due to the lower resolution of the Infinium assays (about 60 times more loci were assessed by RRBS than by the BeadChips, data not shown), also here both MethylCollector kits appear to be more sensitive than the MethylMagnet kit.

### Sensitivity and specificity based on CpG-content of the mapped fragments: the Fragment CpG-plot

**Figure 4:**
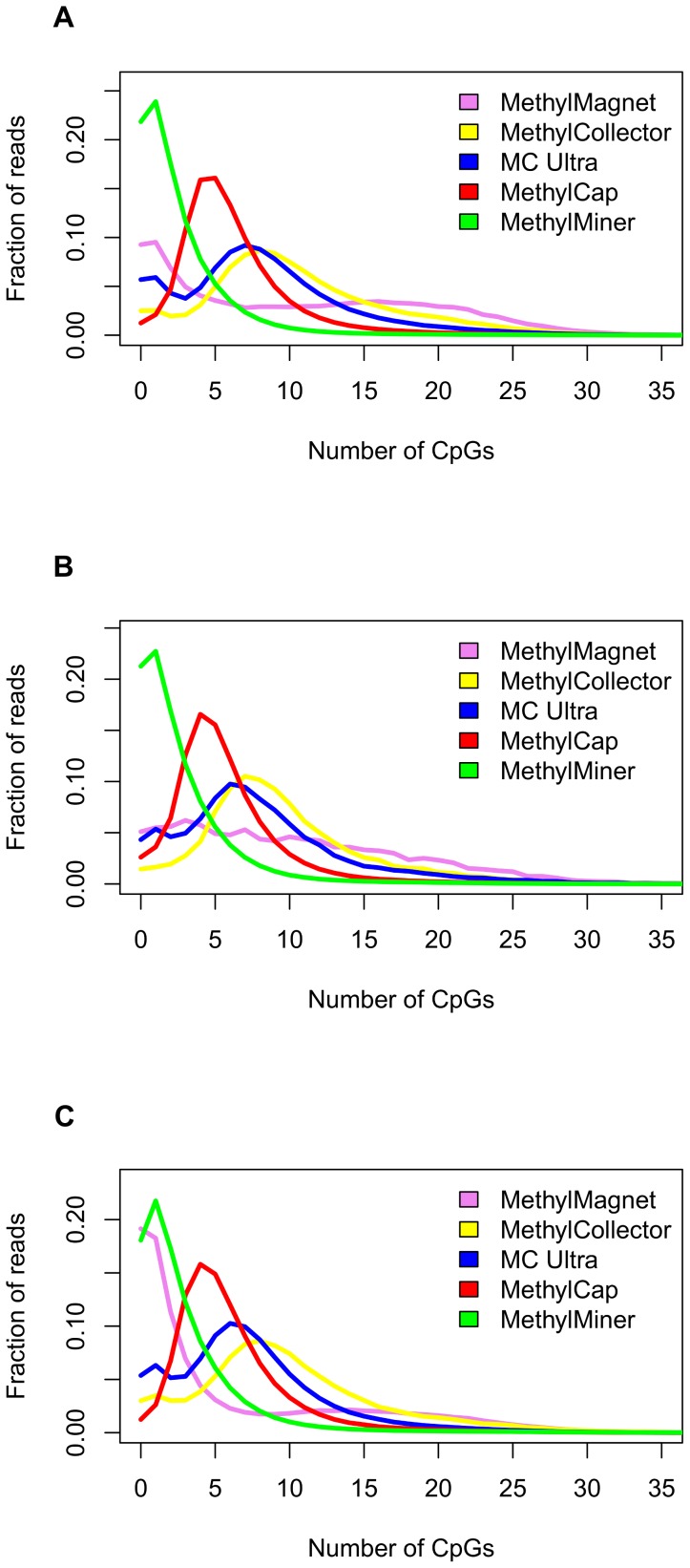
Fragment CpG-plots. Fractions of mapped MBD-seq fragments with different CpG-counts for cell lines HCT15 (A), DU145 (B) and PC3 (C) for the different kits: MethylMagnet (violet), MethylCollector (yellow), MethylCollector Ultra (MC Ultra, blue), MethylCap (red) and MethylMiner (green).

Since MBD kits theoretically only capture methylated cytosines in a CpG-dinucleotide context, the CpG-content profile of the mapped fragments might be used as a proxy for sensitivity and specificity. Figure 4 depicts the percentages of sequences for each kit corresponding with a specific CpG-count for the HCT15 cell line. This type of diagnostic plot, here coined the "Fragment CpG-plot", therefore also adjusts for yield differences. This plot is similar to Figures 2A–C and 3A–B but depicts fractions of reads as a function of CpG-content of captured reads instead of independently assessed methylation degrees. Figure 4 suggests that the MethylCap and MethylCollector kits are featured by the highest specificity, i.e. lowest fraction of non CpG containing fragments, followed by the MethylCollector Ultra and MethylMagnet kit. Also here, the MethylMiner profile exhibits low specificity, but also no additional CpG containing peak, indicating low sensitivity. Sensitivity, *i.e.* detection of loci with low degrees of methylation, can here be approximated as the amount of captured fragments with low (but non zero) amounts of CpGs. The MethylCap kit profiles demonstrate peaks with maxima at around 5 CpGs/sequence, which suggests the highest sensitivity, followed by the MethylCollector, MethylCollector Ultra and MethylMagnet kits where more CpGs were required. Note that for the latter kit, profiles tended to vary between samples. Extremely low yield (DU145, Table 1) or presence of noise fractions not assessed by RRBS and Infinium (PC3) are probable causes.

Overall, however, results are very consistent between Fragment CpG-plot and independent validation data (RRBS, Infinium BeadChips) derived sensitivity and specificity, indicating that the former can indeed be used as a very straightforward tool for quality evaluation.

## Discussion

MBD-seq has a clear potential to become the most widely used methodology for completely genome-wide methylation studies until the advent of more appropriate and particularly cost-efficient sequencing technologies. Several protocols and commercial kits for MBD-based capturing of methylated fragments are currently available, but there is a general lack of independent information on yield, sensitivity and specificity of the different methodologies. In addition, there are no quality diagnostic tools for the identification of aberrant sequencing profiles, cf. the diagnostic plots for microarray quality control [25]. In this study, the quality of commercially available kits was evaluated using external validation data and inherent sequence data characteristics. The results overall confirmed the necessity of quality control.

Yield was a first major factor of difference between kits. It is clear that yield is a crucial parameter as it affects the number of MBD capturing steps (with associated cost) and amounts of required (often valuable) sample to obtain the necessary DNA quantities for high coverage sequencing. Therefore, yield should always be taken into consideration when comparing different kits and experimental conditions. Yield is closely entangled with sensitivity and specificity, complicating the assessment of the latter. Ideally, equal quantities of captured DNA by the kits would have been used for sequencing, which would at least in theory have allowed for a yield independent comparison of the kits' sensitivity and specificity. However, quantities were too low for several kits (Table 1) to ensure that this approach would have been successful. In addition, it should be noted that coverage differences between samples would have been observed anyway due to variable sequencing efficiencies between lanes and samples, implying that some sort of normalization is always required. Therefore, we opted to sequence all of the material obtained for each of the kits, and to use a data-analytical approach that is unbiased by yield differences. Indeed, *relative fractions* corresponding with independently determined methylation degrees or with CpG-content were studied. This approach was very successful as clearly reproducible results were obtained, although extremely low amounts of mapped fragments often resulted in more variable, lower resolution plots that were harder to interpret. Whereas this implies that a minimal coverage per condition is required for quality control, it should be noted that coverages of several 100.000 mapped fragments are certainly sufficient for Fragment CpG-plot assisted quality control, implying relatively low cost for this type of experimental optimization (compared to the typical biomarker studies where millions of fragments are required).

The different analyses identified the MethylCap kit as the overall best kit due to a consistent combination of high yield, sensitivity and specificity. It should be noted that the high sensitivity of the MethylCap kit also allows for capturing of low CpG-density fragments, which have been reported to be biologically more relevant [23], [24]. The MethylMiner kit, with an even higher yield, demonstrates a general lack of specificity with the used settings, although results for this kit will most likely greatly improve by not including the low-salt elution fraction. Both MethylCollector kits are characterized by lower yields and sensitivity than MethylCap. Based on yield and sensitivity, the MethylCollector Ultra kit performs slightly better than the original MethylCollector kit, which is currently no longer available. Finally, even with some aberrant Fragment CpG-plots, it is clear that the MethylMagnet kit was featured by lower yield, sensitivity and specificity. Although validation by an independent laboratory is indispensable, and other salt elution procedures could have a major impact on the obtained data, results indicate that, of the MBD-based kits under study, the MethylCap kit performs best, followed by the MethylCollector kits. Whereas these conclusions are based on two sources of independent validation data, the Fragment CpG-plots allowed us to perform this ranking solely based on the generated sequence data. This demonstrates that the value of this study surpasses the limitations imposed by the restricted set of experimental conditions evaluated.

Interestingly, MethylCap is the only kit for which the MBD originates from MeCP2, while this is MBD2 for the other kits. Although both proteins bind methylated DNA in a very similar manner, they exhibit some sequence specificity (around the ^m^CpG) and MBD2 was reported to show a higher binding affinity than MeCP2 [28], [29]. However, it remains unclear to what extent overall sensitivity and specificity are affected by the fact that only (specific recombinant forms of) the MBD-domains are used in the kits.

As it is costless and straightforward, we suggest to use the Fragment CpG-plot to monitor quality for experimental optimization, but also during standard applications to identify aberrant profiles. Note that (variants of) Fragment CpG-plots have already been used in other studies, e.g. [22], [23], underscoring the intuitive character of these diagnostic plots. However, this is the first study performing an objective evaluation and independent validation of their practical use.

It should be taken into account that there are several limitations to the Fragment CpG-plot. First, longer fragments will (on average) consist of more CpGs, implying that the degree of DNA-fragmentation prior to capturing will affect the Fragment CpG-plots. While the impact is limited for the identification of aberrant profiles, or for an in house experimental optimization (cf. this study), it will certainly complicate comparisons between different studies. To obtain the most reliable Fragment CpG-plots, one should always attempt to obtain fragments as short as possible, as long as it does not affect capturing, sequencing and mapping yields. Since this will also increase the resolution of the sequencing methodology itself, i.e. the exact methylated cytosines can be more accurately identified, this is an objective aim that will results in an overall improvement of the data. A second putative limitation is that Fragment CpG-plots are less suitable for MeDIP-experiments, as the latter will also measure non-CpG-methylation. However, certainly in a human context, non-CpG-methylation is limited: it particularly occurs in embryonic (and other) stem cells, and even in these cells it has been estimated to compose only one quarter of the total amount of methylated cytosins [30]. Therefore, Fragment CpG-plots will most likely also be suitable to identify aberrant MeDIP profiles or to perform comparative MeDIP studies. However, in other species with more prominent non-CpG-methylation, these diagnostic plots might be insufficient. A final limitation is the fact that Fragment CpG-plots cannot be used for short single-end read sequencing data. However, paired-end sequencing is the current standard for enrichment based sequencing experiments as it ensures more accurate mapping. Fragment CpG-plots are also suitable for single-end reads that are adequately long, preferably encompassing the full captured fragment, which will become more important in the future.

In conclusion, DNA-methylation is increasingly gaining importance in clinical practice, both from a diagnostic, prognostic and pharmacogenomic viewpoint. Currently, MBD-based sequencing is the most cost-efficient method for the putative genome wide identification of DNA-methylation. Here, we demonstrated major differences in yield, sensitivity and specificity of commercially available kits, illustrating the need for objective quality measures for this type of experiments. Independent validation is however not always necessary, as Fragment CpG-plots already provide us with a good overview of sensitivity and specificity. Indeed, solely based on this diagnostic plot, it was possible to identify MethylCap as the best kit under the conditions used in this study. Reporting this diagnostic plot, together with yield, facilitates experimental quality evaluation, for comparative studies but certainly also for individual experiments and it might be considered to establish this as a standard practice.
